# Mitochondrion-Dependent Cell Death in TDP-43 Proteinopathies

**DOI:** 10.3390/biomedicines9040376

**Published:** 2021-04-02

**Authors:** Chantal B. Lucini, Ralf J. Braun

**Affiliations:** Research Area Neurodegenerative Diseases, Center for Biosciences, Faculty of Medicine/Dental Medicine, Danube Private University, 3500 Krems an der Donau, Austria

**Keywords:** TDP-43, proteinopathy, mitochondria, mitochondrial permeabilization, mtDNA, apoptosis, cell death

## Abstract

In the last decade, pieces of evidence for TDP-43-mediated mitochondrial dysfunction in neurodegenerative diseases have accumulated. In patient samples, in vitro and in vivo models have shown mitochondrial accumulation of TDP-43, concomitantly with hallmarks of mitochondrial destabilization, such as increased production of reactive oxygen species (ROS), reduced level of oxidative phosphorylation (OXPHOS), and mitochondrial membrane permeabilization. Incidences of TDP-43-dependent cell death, which depends on mitochondrial DNA (mtDNA) content, is increased upon ageing. However, the molecular pathways behind mitochondrion-dependent cell death in TDP-43 proteinopathies remained unclear. In this review, we discuss the role of TDP-43 in mitochondria, as well as in mitochondrion-dependent cell death. This review includes the recent discovery of the TDP-43-dependent activation of the innate immunity cyclic GMP-AMP synthase/stimulator of interferon genes (cGAS/STING) pathway. Unravelling cell death mechanisms upon TDP-43 accumulation in mitochondria may open up new opportunities in TDP-43 proteinopathy research.

## 1. Introduction

### 1.1. TDP-43 Structure, Function, and Localization

Trans-active response element (TAR) DNA binding protein-43 (TDP-43) is a 414 amino acid long protein encoded by the TAR-DNA binding protein gene (*TARDBP*) on chromosome 1. It was found in the 1990s as a repressor protein associated with HIV-1 transcription, binding the TAR DNA sequence of the viral genome and regulating the viral gene expression [1]. The N-terminal region harbors a nuclear localization signal (NLS) followed by two RNA recognition motifs (RRM1 and RRM2) and a nuclear export signal (NES) [2]. TDP-43 is predominantly localized in the nucleus, where it serves as an RNA/DNA binding protein preferentially recognizing UG/TG-rich motifs of RNA and DNA [3,4,5,6]. In- and outside of the nucleus, TDP-43 regulates mRNA splicing, translation, transport, and degradation [7,8]. TDP-43 binds to more than 6000 mRNAs, which corresponds to nearly 30% of the transcriptome [9,10]. The C-terminal region has a prion-like glutamine/asparagine-rich domain and a glycine-rich region, which is highly aggregation-prone [11]. In addition, there are three internal mitochondrial localization motifs (M1, M3, M5), which consist of continuous stretches of hydrophobic amino acids [12]. These motifs enable the import of TDP-43 into the mitochondrial matrix. The mitochondrial localization of TDP-43 has been initially associated with pathological outcome [12], but physiological roles have also been discussed (see below). Thus, TDP-43 is an RNA/DNA-binding protein, which can be found in all compartments that contain RNA or DNA, i.e., the nucleus, the cytosol, and the mitochondria.

### 1.2. TDP-43 and Neurodegeneration

In 2006, TDP-43 was found as a major component of insoluble and ubiquitinated inclusions in the brains of patients suffering from amyotrophic lateral sclerosis (ALS) and frontotemporal dementia (FTD) [13]. Since then, more diseases were classified as TDP-43 proteinopathies, including limbic-predominant age-related TDP-43 encephalopathy (LATE) [14], and inclusion body myositis (IBM) [15].

In 90% of ALS and 50% of FTD cases, TDP-43 was depleted from the nucleus and concomitantly accumulated in the cytoplasm, where it formed aberrant protein aggregates or localized to cellular membranes, including mitochondria [13,16,17,18,19]. Besides full-length TDP-43, 25–35 kDa C-terminal TDP-43 fragments and post-translationally modified TDP-43 species enriched in the cytoplasm in a pathological context [20]. Common disease-associated post-translational modifications are hyperphosphorylation, ubiquitination, acetylation, poly ADP-ribosylation (PARylation), and cysteine oxidation. All of them modulate the formation of cytoplasmic aggregates and the cellular localization and function of TDP-43 [2].

Whereas in the vast majority of ALS and FTD cases with TDP-43 pathology the *TARDBP* gene remains unaltered, mutations in *TARDBP* are associated with familiar and sporadic forms of ALS and FTD [21,22,23,24,25]. These mutations were predominantly found in gene regions, encoding the aggregation-prone C-terminal domain of TDP-43 [11]. Many of these mutations enhance the intrinsic property of TDP-43 for aggregation and influence its subcellular localization, including to mitochondria. Mutations encoding the RNA-recognition motifs could also be identified, suggesting a role of altered RNA binding in the pathogenesis triggered by hereditary and sporadic TDP-43 disease variants [17].

Both loss- and gain-of-function mechanisms are discussed to contribute to pathology in TDP-43 proteinopathies. Depletion of nuclear TDP-43 affects the nuclear functions of TDP-43. Cytoplasmic truncation, post-translational modifications, misfolding, and aggregation may play a dual role: it affects the physiological functions of soluble full-length TDP-43 in this compartment, but may also lead to the generation of various cytotoxic TDP-43 species. Aberrant TDP-43 species interfered with stress granule assembly, the ubiquitin-proteasome system, autophagy, the endo-lysosomal pathway, vesicular transport, and the transport of macromolecular complexes through the nuclear pores [26,27,28,29,30,31,32,33]. Due to the diverse effects of aberrant TDP-43 species, it is not surprising that disease-associated mitochondrial dysfunction may also be triggered in TDP-43 proteinopathies and TDP-43 model systems.

### 1.3. TDP-43 and Mitochondria in Neurodegeneration

Since neurons rely on intact mitochondrial function for their energy metabolism and neuronal activity [34], mitochondrial defects could aggravate neurodegeneration [35]. In material from ALS and FTD patients with TDP-43 pathology, and in numerous TDP-43 disease models (yeast, cell culture, mouse, flies), either expressing wild-type TDP-43, disease-associated TDP-43 variants, TDP-43 fragments, or with TDP-43 knock-down, hallmarks of mitochondrial damage and dysfunction were observed. This included mitochondrial membrane permeabilization, loss of mitochondrial membrane potential, generation of mitochondrial ROS, and decreased OXPHOS activities [12,19,36,37,38]. In only a few studies, no alterations in mitochondrial function and bioenergetics were explicitly described upon TDP-43 expression [39,40]. Thus, mitochondrial dysfunction may be critical for cytotoxicity in TDP-43 proteinopathies, which was proposed in many studies [12,19,37,38,41,42,43]. However, the underlying molecular mechanisms may be multiplex and depend on the cellular level of TDP-43, its localization, and modification.

Some mutations in *TARDBP* may affect the regulation of mRNAs coding for proteins involved in mitochondrial physiology. This was shown for the differential splicing of the Mitochondrial fission regulator-1 (Mtfr-1) pre-mRNA in cells expressing the disease-associated TDP-43-M337V variant [44]. Mitochondrial damage may also be triggered by the interaction or loss of interaction of nuclear TDP-43 with transcription factors, such as Forkhead box protein O3 (FOXO3a). FOXO3a attenuates transcription of many genes with mitochondrial function. Nuclear TDP-43 inhibited FOXO3a, and therefore, enabled the transcription of those genes. Consequently, nuclear depletion of TDP-43 attenuated transcription of mitochondrial genes, culminating in mitochondrial dysfunction [45].

The vast majority of mitochondrial proteins is encoded in the nuclear DNA, translated as precursors in the cytosol and post-translationally imported into mitochondria. Cytosolic TDP-43 may also interact with cytosolic chaperones responsible for protein import into mitochondria. Here, mitochondrial damage would occur due to lowering of nuclear-encoded mitochondrial protein levels [45].

Mitochondrial TDP-43 species were found in various disease model systems and in neurons of post mortem material from ALS patients [12,36,37,43,46,47,48,49,50,51]. The TDP-43 accumulation in mitochondria in a disease context suggests a pathological function of mitochondrial TDP-43. However, physiological roles for the mitochondrial localization of this protein have also been proposed [36,52]. Before discussing how TDP-43 is involved in mitochondrion-dependent cell death, we, therefore, critically compare the evidence for the physiological and pathophysiological roles of mitochondrial TDP-43.

## 2. Physiological Roles of Mitochondrial TDP-43

**Figure 1:**
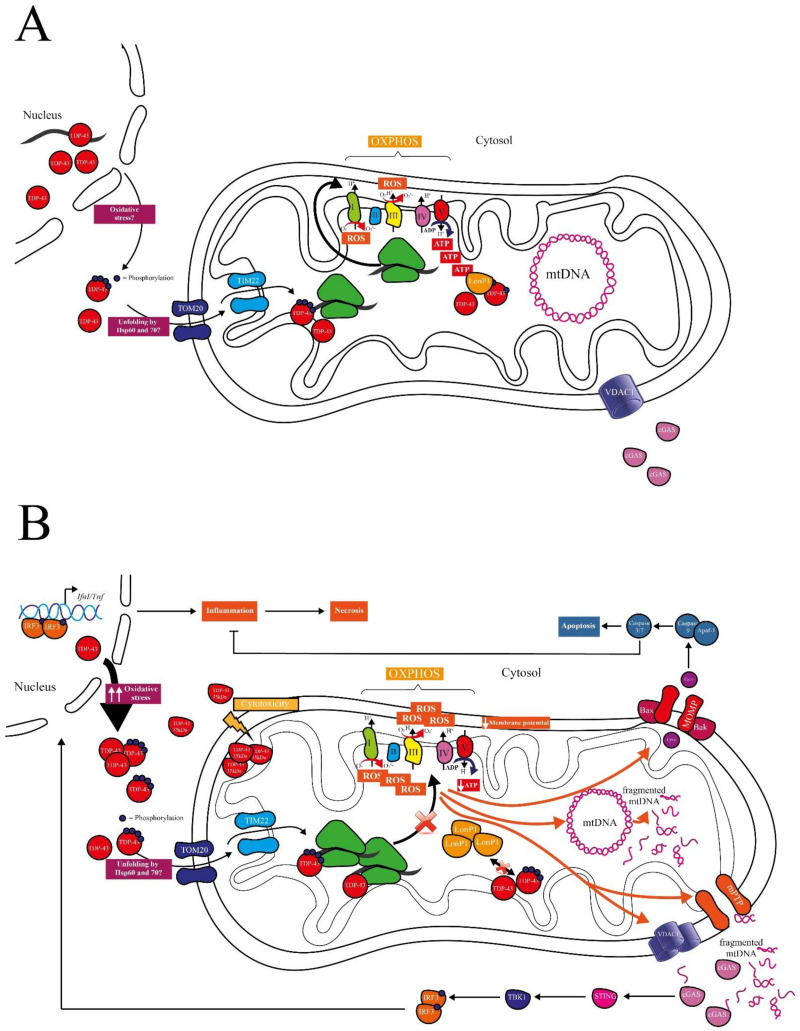
Potential physiological and pathophysiological roles of TDP-43 in mitochondrial function and cell death. (**A**) Physiological roles: Oxidative stress triggers the export of nuclear TDP-43 into the cytosol. Exported and newly synthesized cytosolic TDP-43 might be phosphorylated. Upon unfolding by Hsp60 or Hsp70, phosphorylated and unphosphorylated TDP-43 might be imported into mitochondria through TOM20 and TIM22. TDP-43 localizes in the matrix-facing part of the mitochondrial inner membrane (MIM), where it might regulate expression of OXPHOS components. Surplus, misfolded, or aberrantly post-translationally modified TDP-43 might also be imported from the cytosol into mitochondria for its degradation by LonP1, protecting the cytosol from aggregation-prone proteins. (**B**) Pathophysiological roles: Chronically increased oxidative stress (two arrows tending upwards) increases nuclear depletion and cytosolic accumulation of TDP-43 followed by its import into mitochondria. The truncated 35 kDa form is also imported in the mitochondria but is stuck in the intermembrane space and its cytotoxicity is not related to mt-RNA binding. Elevated TDP-43 levels in the mitochondria increases TDP-43-binding on mt-mRNAs of several OXPHOS components, affect the mitochondrial respiratory chain (red diagonal cross), leading to lower ATP production, decreased mitochondrial membrane potential, and increased ROS. Decreased ATP hinders TDP-43 degradation in the mitochondria via LonP1 (red diagonal cross). Mitochondrial dysfunction leads to the opening of mPTP and oligomerization of VDAC1, and consequently, fragmented mtDNA is released in the cytosol and sensed by cGAS. cGAS binds to mtDNA and activates STING, which triggers the expression of proinflammatory genes through the IFN pathway, culminating in necrotic cell death. Alternatively, cell death may be caused through the canonical intrinsic apoptosis pathway. Here cytochrome *c* is released from mitochondria in a MOMP- and Bax-dependent manner to activate caspase-9 and the caspase cascade. This includes caspases-3/7, which attenuate inflammation (bar-headed arrow). IFN: interferon; LonP1: Lon protease; MOMP: mitochondrial outer membrane permeabilization; mPTP: mitochondrial permeability transition pore; OXPHOS: oxidative phosphorylation.

TDP-43 bears three mitochondrial localization motifs, which make it plausible to speculate for physiological functions of TDP-43 in mitochondria [12]. TDP-43 entered the mitochondrial matrix through the translocase of the outer membrane (TOM20) and the translocase of the inner membrane (TIM22) [12,38]. Unfolding of the protein was necessary to expose the import signals, which are otherwise hidden in the correctly folded protein. There are several hypotheses in this regard involving Hsp60 and Hsp70 [53], but the exact mechanism remains unresolved (Figure 1A).

Izumikawa and colleagues proposed that mitochondrial TDP-43 may be required for maintaining appropriate levels of at least certain mitochondrial (mt)-RNAs and for maintaining correct mitochondrial function (Figure 1A) [36]. siRNA-mediated TDP-43 knockdown in the human breast cancer cell line MCF7 reduced the mitochondrial membrane potential, the cellular levels of ATP, and the enzymatic activity of electron transfer complex I compared to control. The authors also showed some reduction in 16S rRNA as well as in cytochrome *c* oxidase I (COX1) mt-mRNA. Nevertheless, there is a lack of information if these effects upon TDP-43 knockdown are due to the lack of TDP-43’s mitochondrial localization or a general loss-of-function of TDP-43, indirectly interfering with mitochondrial function.

Termsarasab and colleagues observed that endogenous TDP-43 is highly phosphorylated in mitochondria of the pontine nuclei, thalamus, Cornu Ammonis 3 (CA3) region of the hippocampus, and orbital cortex of young and normal aged mice without any evidence of neurodegenerative or systemic disorders [52]. Through high-resolution double fluorescence images and confocal microscopy, they revealed that mitochondrial TDP-43 localization in these brain regions was paralleled to nuclear depletion and cytoplasmic accumulation of this protein. The authors speculated that TDP-43’s distribution pattern contributes to some physiological functions peculiar of these brain regions, such as speech and swallowing, or alternatively reflects an early pre-pathological state. Unfortunately, the authors were just able to analyze six male mice per group and were not able to obtain older animals, leaving room for further research.

One step towards unravelling one of the potential physiological or pre-pathological functions of TDP-43’s mitochondrial localization was the observation that TDP-43 induced the mitochondrial unfolded protein response (UPR^mt^) in T-Rex293 cells and in *Drosophila* expressing recombinant wild-type and mutant (A315T) TDP-43 [37]. One of the major players of UPR^mt^ was the Lon protease 1 (LonP1), which degraded TDP-43 in vitro. LonP1 interacted with TDP-43 and reduced the mitochondrial TDP-43 protein levels in T-Rex293 cells and in *Drosophila*. The authors proposed a model where the mitochondrial damage by TDP-43 triggered the activation of LonP1. Another more physiological scenario could be that cytosolic TDP-43, misfolded or aberrantly post-translationally modified, is imported into mitochondria for degradation by LonP1 (Figure 1A). Interestingly, a similar mechanism of mitochondrion-mediated proteolysis for degradation of misfolded cytosolic proteins was recently found in yeast, the “mitochondria as guardian in cytosol” (MAGIC) system. One of the major MAGIC players was Pim1, the homologous yeast protease to LonP1 [50]. Under heat shock, human TDP-43 was imported into yeast mitochondria in this study, although no data showed whether human TDP-43 was degraded via yeast Pim1 [50]. These observations suggest that the import of surplus, misfolded, or aggregation-prone cytosolic proteins into mitochondria for their degradation is evolutionary conserved, and a further physiological quality control mechanism ensuring cellular proteostasis.

Thus, despite the presence of mitochondrial localization motifs, physiological roles of mitochondrial TDP-43 on mitochondrial function remain speculative. Whether mitochondrial import of TDP-43 is a physiological mechanism to prevent its aberrant accumulation in the cytosol also needs further investigation.

## 3. Pathological Roles of Mitochondrial TDP-43

Studies showing the mitochondrial localization of TDP-43 predominantly dealt with disease cases or disease models, supporting the pathological roles of mitochondrial TDP-43. Sasaki and colleagues found mitochondrial TDP-43 in patients with sporadic ALS [47], and Hong and coworkers observed mitochondrial localization of both full-length TDP-43 and its C-terminal fragments in NSC-34 mouse motor neuron-like cells [49] (Figure 1B). In a groundbreaking work, Wang et al. confirmed the co-localization of TDP-43 with mitochondria in spinal cord and frontal cortex tissue of ALS and FTD patients, as well as, in lower abundance, in age-matched controls [12]. They further showed TDP-43’s mainly sub-localization in the matrix-facing mitochondrial inner membrane (MIM), as confirmed by immuno-electron microscopy. The authors also demonstrated that mitochondrial localization increased with disease-associated TDP-43 variants (G298S and A382T) in fibroblasts derived from patients compared to age-matched control. They confirmed these findings in HEK293 cells and brain and spinal cord tissue samples from transgenic mice overexpressing human wild-type or disease-associated TDP-43. Inside mitochondria, TDP-43 interacted with several mitochondrial mRNAs (mt-mRNAs) (Figure 1B), comprising those encoding complex I subunits, namely NADH-ubiquinone oxidoreductase chains 3, 5, and 6 (ND3, ND5, ND6), two of the three complex IV subunits (COXI, COXIII), one complex III subunit (cytochrome *b*, CYTB), and subunit 6 of ATP synthase (A6) in isolated brain mitochondria of mice overexpressing human wild-type or disease-associated TDP-43. TDP-43 overexpression directly impaired the loading of mitochondrial ribosomes onto mRNAs encoding for ND3 and ND6. These effects were corroborated in HEK293 cells overexpressing wild-type or disease-associated TDP-43 and in tissues from ALS-spinal cords or FTD cortices. Here, the levels of ND3 and ND6 were specifically reduced, whereas other OXPHOS proteins remained unaltered. Consequently, disassembly and dysfunction of complex I was observed, leading to reduced mitochondrial membrane potential, oxygen-consumption rate, and ATP levels (Figure 1B) accompanied by mitochondrial fragmentation. Notably, all effects seen on mitochondrial expression and function were much stronger in the disease-associated TDP-43 variant than in wild-type. Inhibition of TDP-43’s mitochondrial localization with the selective inhibitor PM1 rescued ND3 and ND6 expression and restored mitochondrial function. The inhibitor was able to reverse motor-coordinative and cognitive dysfunction in the transgenic mouse model expressing the disease-associated human TDP-43-M337V variant [51]. These findings support the pathological role of mitochondrial localization of specific disease-associated TDP-43 variants. Whether these effects are also relevant for wild-type TDP-43 remained unclear. However, this is of interest, as in the vast majority of ALS and FTD cases with TDP-43 pathology *TARDBP* is not mutated.

In 2017, Izumikawa and colleagues observed that exogenous overexpression of wild-type TDP-43 in the human T-Rex 293 cell line also resulted in mitochondrial localization of this protein [36]. Here, TDP-43 stabilized specific mt-tRNAs and mt-mRNAs, probably through direct binding and complementary double-strand formation. Binding to mt-RNAs may affect mitochondrial translation (Figure 1B). Consequently, the authors observed reduced levels of two complex I subunits (ND2, ND5), a complex III subunit (CYTB), and an ATP synthase membrane subunit 8 (ATP8). As a result of overexpression of wild-type TDP-43, the authors measured increased ROS, reduced OXPHOS, and inhibition of cell proliferation. Notably, they observed that inhibition of mitochondrial transcription with ethidium bromide rescued cell proliferation. This may suggest that imbalance of mitochondrial translation triggered by TDP-43 binding to mt-mRNAs contributes to TDP-43-triggered cytotoxicity.

The observations by Wang and colleagues and Izumikawa and co-workers were replicated by Salvatori et al., who analyzed NSC-34 mouse motor neuron-like cells overexpressing full-length or truncated 35 kDa TDP-43 [43]. Interestingly, they observed an increased mitochondrial localization for the truncated form (Figure 1B), which remained stuck in the intermembrane space, as it lacks the M1 mitochondrial localization sequence. Consequently, the 35 kDa TDP-43 fragment was not able to bind to mtRNA, which was localized in the mitochondrial matrix, and did not cause mitochondrial destabilization. They suggested that the potential cytotoxicity of truncated TDP-43 was probably caused by other cellular mechanisms, such as the generation of large cytoplasmic aggregates [54] and not due to interference with mitochondrial function. These results were slightly in contrast to what was seen in the same cell model before, where both forms, the full-length and 35 kDa TDP-43 fragment, caused mitochondrial dysfunction [49], leaving some doubt about the potential pathological role of the truncated form in mitochondria.

Reduced mitochondrial ATP synthesis due to suppressed complex I activities, reduced mitochondrial membrane potential, and increased ROS, was proposed to result in the observed severe structural deformations of the mitochondria in patients with TDP-43 proteinopathy, as well as in HEK293 cells with induced exogenous TDP-43 expression (wild-type and A315T variant) and in transgenic flies (wild-type and A315T variant) [37]. Remarkably, mitochondrial localization was seen in both TDP-43 and control groups, confirming that TDP-43 is also localized in mitochondria in a non-pathological context. However, a higher percentage was found in TDP-43 proteinopathy patients, TDP-43 expressing cell lines, and transgenic flies.

Reduced ATP synthesis driven by TDP-43 may have an additional deleterious effect: the previously described mitochondrial protease LonP1, which interacts with TDP-43 and eventually degrade it, is ATP-dependent. This could lead to a vicious cycle, where TDP-43 accumulates in the mitochondria, affecting ATP synthesis, thereby interfering with its own degradation via the LonP1 protease (Figure 1B). Despite the upregulation of the UPR^mt^, which has been observed in TDP-43 proteinopathy patients, in both cell line and transgenic flies expressing TDP-43, LonP1 would be incapable of degrading pathological mitochondrial TDP-43 [37]. This could ultimately trigger mitochondrial damage and mitochondrion-dependent cell death. Before discussing the roles of TDP-43 in regulated cell death, we first outline the role of mitochondria in apoptotic and necrotic cell death.

## 4. The Role of Mitochondria in Intrinsic Apoptosis and Inflammatory Cell Death

Mitochondria play a central role in orchestrating many subroutines of regulated cell death (RCD), including non-inflammatory intrinsic apoptosis and inflammatory necrotic cell death. One way by which mitochondria initiate cell death is through mitochondrial outer membrane permeabilization (MOMP). MOMP nearly always leads to cell death and is, therefore, tightly regulated through the Bcl-2 superfamily, comprising pro-apoptotic, anti-apoptotic, and death effector proteins. In intrinsic apoptosis, once activation of the cell death effectors Bax and Bak is triggered, cytochrome *c* is released in the cytosol and binds Apoptotic protease activating factor-1 (Apaf-1), recruiting caspase-9 to form the apoptosome, which leads to the activation of the executioner caspases-3 and -7 [55,56]. The activation of apoptotic caspases has an immunosilencing effect during cell death, as caspases can directly cleave components of the inflammatory pathway and suppress protein translation (Figure 1B). However, MOMP can also induce inflammation by activation of the cGAS/STING pathway and pro-inflammatory Nuclear Factor-kappa-B (NF-κB) signaling. In this case, mtDNA and not yet defined mitochondrial proteins are released through Bax/Bak macropores subsequent to MOMP formation, triggering pro-inflammatory signaling, resulting in the release of inflammatory cytokines and ultimately in cell death [57,58].

Intrinsic apoptosis and inflammatory cell death can also be mediated by prolonged opening of the mitochondrial permeability transition pore (mPTP) in response to oxidative stress [59]. The structure of mPTP is still under debate. Cyclophilin D (CypD) is so far the only protein that has been shown to be critical for mPTP. Other putative components of the mPTP are F_1_F_0_ ATP synthase, Adenine nucleotide translocator (ANT), and mitochondrial Phosphate Carrier (PiC) [60]. Some reviews [60,61] and research publications have claimed that Voltage-dependent anion-selective channel-1 (VDAC1) is not part of the mPTP [62,63]. Other publications have shown the opposite [64,65,66]. However, they all agree that VDAC1’s function is tightly correlated to mPTP. It plays a central role in releasing mitochondrial ROS (mROS) from the intermembrane space [67], and VDAC oligomers interact with mtDNA to release it into the cytosol [68]. mROS release in the cytosol can cause nuclear DNA damage, triggering pro-apoptotic signaling [60], whereas cytosolic presence of mtDNA can activate the cGAS/STING signaling leading to inflammatory cell death [69].

cGAS is a cytosolic DNA sensor, playing a major role in the innate immune response by binding foreign and self-DNA. Binding of DNA triggers the production of cGAMP, which activates STING, which in turn recruits and activates Serine/threonine-protein kinase-1 (TBK1). TBK1 phosphorylates the transcription factor Interferon regulatory factor-3 (IRF3), which dimerizes, translocates in the nucleus, and activates the type I interferon response. The activation of the cGAS/STING pathway has also been brought in relationship with increased necroptosis through a Tumor necrosis factor (TNF)-dependent mechanism [70] (Figure 1B). The cGAS/STING pathway also plays a role in cell senescence. Accumulated oxidative damage to mitochondrial membrane proteins and lipids in senescent cells increases membrane permeability, leading to membrane break-up [69,71]. Here, the binding of cGAS to mtDNA activates the expression of NF-κB and triggers secretion of senescence-associated secretory phenotype (SASP) factors, including Interleukine-6 (IL-6), C-X-C motif chemokine 10 (CXCL10), TNF-α, and several other chemokines. However, the connection between SASP regulation and the cGAS/STING pathway is still largely unknown [69].

## 5. TDP-43’s Role in Mitochondrion-Dependent Apoptosis and Inflammatory Cell Death

In yeast expressing human TDP-43, a critical interplay between mitochondria, mtDNA, and TDP-43-mediated regulated cell death was already described in 2011 [41], which was later confirmed [72]. TDP-43 aggregates were found peri-mitochondrially and TDP-43 expression resulted in decreased cell survival, increased mitochondrial ROS generation, and enhanced plasma membrane permeability in a dose- and age-dependent manner [41]. TDP-43-triggered cytotoxicity culminated in both apoptosis and necrosis. Notably, cell death in yeast increased proportionally to the cellular’s mtDNA content and OXPHOS activity [41]. Respiratory activity clearly enhanced TDP-43 toxicity in yeast, but it is probably not the only mechanism responsible for cell death [72]. Whether part of the observed peri-mitochondrial TDP-43 was imported into mitochondria and in the MIM fraction, as observed in higher TDP-43 proteinopathy model systems [12,37,38], was not analyzed in these studies dealing with TDP-43-triggered yeast cell death [41,72]. However, Ruan et al. observed that upon heat shock, a fraction of human TDP-43 could be imported into yeast mitochondria, but these authors did not analyze whether this TDP-43 fraction was cytotoxic [50]. In a recent paper, TDP-43-triggered cell death in yeast was linked to Cyclin C, the mitochondrial fission factor Dynamin-related protein 1 (Dnm1), and Ybh3, the yeast homolog of human pro-apoptotic BH3-containing factors, such as Bax [73]. Cyclin C has previously been described to translocate from the nucleus in the cytoplasm to trigger Dnm1- or Drp1-dependent mitochondrial fission and BH3- or BAX-dependent mitochondrial permeabilization in yeast and mammalian cells [74,75,76]. Therefore, it is likely that human TDP-43 triggers mitochondrial permeabilization in yeast. Deletion of genes encoding for the mitochondrial cell death-mediators apoptosis-inducing factor 1 (Aif1p), endonuclease G (Nuc1p), and cytochrome *c* did not rescue TDP-43-triggered clonogenic cytotoxicity in yeast [41]. Therefore, it is unlikely that the release of one of those factors from mitochondria into the cytosol is decisive for execution of regulated yeast cell death upon TDP-43 expression. Further research is needed to elucidate whether intra- or extramitochondrial TDP-43 species trigger mitochondrion-dependent cell death in yeast, whether MOMP- or mPTP-related mitochondrial permeabilization occurs, and which mitochondrial factor is released from yeast mitochondria for execution of cell death. Based on the importance of mtDNA content for TDP-43-triggered cytotoxicity, and the recent findings in mammalian cells (see below), it is tempting to speculate that this factor might be mtDNA.

As mentioned before, Wang and colleagues described the vicious cycle of TDP-43 accumulation in the mitochondria, ATP depletion, and therefore, inability of ATP-dependent LonP1 to clear up the mitochondrion from too much TDP-43 [37]. Ultimately, this would lead to cell death. The authors showed a significantly higher but small extent of apoptosis by Annexin V-positive/PI-negative staining 36 h post induction in the TDP-43 cell model compared to control. The effects are moderate, potentially because mitochondrial dysfunction is an early event preceding TDP-43-triggered cell death. The cell death routines involved could not be dissected in this study, because just a small part of the dead transgenic rat primary neurons expressing TDP-43 wild-type or mutant were caspase-3 positive. Patient-derived induced pluripotent stem cells (iPSC) showed no significant difference in cell death, probably because age or age-associated phenotypes are usually lost after fibroblast reprogramming [77,78]. Other molecular modulators than the classical apoptotic pathway may also play a role, such as cGAS/STING, sensing mtDNA leakage in the cytosol and triggering an inflammatory response [38].

TDP-43 proteinopathies, such as ALS, are often associated with inflammation with the release of NF-κB-related cytokines and elevated type I interferon response. In mouse models, these inflammatory signals were seen before onset of the symptoms of the disease, suggesting that they play a role in disease pathogenesis. Whether the cGAS/STING pathway is involved in inflammation in TDP-43 proteinopathies has not been elucidated until recently [38]. The activation of the cGAS/STING signaling by TDP-43 occurs because of mtDNA release in the cytosol (Figure 1B). In detail, TDP-43 was shown to be imported into mitochondria through the mitochondrial translocase of the inner membrane TIM22. Inhibiting the mitochondrial import of TDP-43 with the competitive inhibitor PM1 prevented leakage of mtDNA in the cytosol in ALS patient iPSC-derived motor neurons. These findings establish a connection between mitochondrial TDP-43 localization and mitochondrial damage. Indeed, the authors observed hallmarks of mitochondrial destabilization, such as loss of the mitochondrial membrane potential and upregulation of mitochondrial ROS. The inhibition of ROS production prevented inflammation in response to TDP-43. The authors further proved that inhibition of VDAC-1 oligomerization prevented cytosolic accumulation of mtDNA and inflammation in ALS patient iPSC-derived motor neurons. In addition, deletion of VDAC-1 in TDP-43 overexpressing mouse embryonic fibroblasts (MEFs) prevented expression of inflammatory genes, such as Interferon beta 1 (*Ifnb*1) and Tumor necrosis factor (*Tnf*). Downstream of these events, it was shown that in response to TDP-43 (wild-type, A315T, or Q331K) overexpression in HEK293T cells, cGAS was bound to mtDNA, leading to activation of STING, which upregulated NF-κB and type I interferon pathways. Similarly as seen in yeast [41], mtDNA depletion decreased the detrimental effects. The authors could not observe any evidence of cleaved caspase-3 in mouse MEFs expressing TDP-43 (wild-type or Q331K) and confirmed that in their model, pro-apoptotic Bax/Bak were not involved in TDP-43-dependent mtDNA release.

In contrast, other groups reported an involvement of the canonical intrinsic apoptotic pathway [79,80] (Figure 1B). Suzuki et al. observed increased cell death in mouse NSC34 motor neuronal and primary cortical neurons with a moderate overexpression of human TDP-43 (two to five times above endogenous level) [79]. In this case Bim, a pro-apoptotic member of the Bcl-2 superfamily, was upregulated and Bcl-xL, an anti-apoptotic factor inhibiting Bax/Bak pore formation, was downregulated. Similarly, Vogt and colleagues examined the effect of human wild-type and mutant (A315T) TDP-43 overexpression targeting neuronal progenitor by in utero electroporation in mice [80]. Both TDP-43 variants induced cell death and caspase-3 activation, which was not observed if p53 was either deleted or pharmacologically inhibited. Similar results were also obtained in human iPSC-derived cortical neurons, where upregulation of pro-apoptotic genes was also observed.

Thus, TDP-43 triggers mitochondrion-dependent cell death in yeast and mammalian cell and animal systems. Both anti-inflammatory apoptosis and pro-inflammatory necrosis can be observed, and the mtDNA plays a central role in regulated cell death.

## 6. Conclusions and Outlook

TDP-43 is a disease-associated RNA/DNA-binding protein, which localizes to the nucleus, the cytosol, and the mitochondria. In this review, we focused on potential physiological and pathological roles of mitochondrial TDP-43, how TDP-43 may trigger regulated cell death with a pivotal contribution of mitochondria, and in which way mt-RNA and mtDNA are involved in these processes (Figure 1).

Whether TDP-43 does have physiological functions in mitochondria is still unknown. As TDP-43 bears mitochondrial localization motifs [12] and as it is localized to mitochondria in non-pathological contexts [37,52], it is tempting to speculate that TDP-43 binds to mtDNA-encoded mRNAs, thereby regulating mitochondrial protein expression [36] (Figure 1A). In such a scenario, mitochondrial TDP-43 modulates mitochondrial functions, such as OXPHOS, in a physiological context. However, further experimental approaches are needed to substantiate this hypothesis. It will be necessary to identify the origin and the subtype of TDP-43 species that enter the mitochondria, and the physiological conditions under which the translocation occurs. For instance, TDP-43 could be depleted from the nucleus, e.g., upon oxidative stress, before its translocation to mitochondria, or a proportion of newly synthesized TDP-43 could directly enter the organelle. It will be of interest whether post-translational modifications are needed to demask the mitochondrial localization motifs, enabling the chaperone-dependent mitochondrial import via the translocases of the outer and inner membranes (TOM and TIM). Since phosphorylated TDP-43 was found to co-exist in mitochondria of healthy mice, phosphorylation patterns could play a crucial role here [52]. In an alternative but non-exclusive scenario, mitochondria could act as a sink, enabling the import of cytosolic TDP-43 species to protect the cytosol from the accumulation of detrimental TDP-43 species (Figure 1A). Here, TDP-43 could be degraded via mitochondrial proteases such as LonP1, which has already been shown to clear mitochondrial TDP-43 [37].

The pathological roles of mitochondrial TDP-43 are better understood. However, similar questions remain unanswered. Which subtype of TDP-43 species enters the mitochondrion, and where does it come from? Here, besides full-length TDP-43, truncated TDP-43 species may also enter the mitochondrion, exerting specific subtypes of cytotoxicity (Figure 1B). Is the import of TDP-43 into mitochondria unintended, and a mere consequence of its cytosolic accumulation? Does the import start as a physiological mechanism to protect the cytosol, but upon overloading of this mechanism it becomes detrimental for mitochondrial functionality and cellular fitness? Nevertheless, pieces of evidence indicate that excessive mitochondrial TDP-43 disturbs mitochondrial protein expression via aberrant binding of mtRNAs, affecting OXPHOS and ATP production, and triggering the production of mitochondrial ROS (Figure 1B) (e.g., [12]). These effects promote regulated cell death via non-inflammatory apoptosis and pro-inflammatory necrosis. It remains unclear which factors determine the induction of these distinct cell death subroutines. Does it depend on the cell type, the level or subtype of cellular stress, the amount of aberrant TDP-43 in mitochondria, or is it an interaction among distinct cytotoxic TDP-43 species within the mitochondrion and/or the cytosol? The release of mtDNA into the cytosol triggers cGAS/STING-dependent inflammation [38]. mPTP and VDAC1 pores are involved in the release of mtDNA, whereas the release of pro-apoptotic cytochrome *c* has been described as effect of mitochondrial outer membrane permeabilization (MOMP) (Figure 1B). It would be of interest whether facilitating MOMP would prevent inflammatory cell death. The general integrity of mtDNA, which decreases upon aging, could also be a potent determinant for whether necrosis and inflammation occurs.

Elucidating the physiological and pathological roles of mitochondrial TDP-43 species and their effects on cell survival and cell death will be a challenging task. The cells continuously integrate functional and toxic signals from a variety of TDP-43 species from different compartments, including the nucleus, the cytosol, and potentially other membranous compartments and organelles. However, it will markedly contribute to a better understanding of molecular pathways underlying TDP-43 proteinopathies, and potentially identify novel targets for pharmacological interventions.

## Data Availability

Not applicable.
